# Supplementary material for: Diagnosing and treating leprosy in a non-endemic setting in a national centre, London, United Kingdom 1995–2018
Source: PLoS Negl Trop Dis. 2022 Oct 20;16(10):e0010799. doi: 10.1371/journal.pntd.0010799 (PMC9624405; doi:10.1371/journal.pntd.0010799)
Supplement: Attachment — Submitted filename: PNTD-D-22-00066 response Reviewers .docx [file pntd.0010799.s001.docx]

PNTD-D-22-00066

**Diagnosing and treating leprosy in a non-endemic setting in a national centre, London, United Kingdom 1995-2018**

2022.07. 13

**Reviewer's Responses to Questions**

**Letter to the editor**

**Thank you for these positive reviews.**

**We have looked at all the points raised. We have responded to the points raised here. Where necessary we have amended the text and attach the revised version with changes shown in Track Changes.**

In this document black text is response to the reviewers points, red will go in the revised version

<b>Key Review Criteria Required for Acceptance?</b></br></br>
As you describe the new analyses required for acceptance, please consider the following:</br></br>

<b>Methods</b></br></br>
-Are the objectives of the study clearly articulated with a clear testable hypothesis stated?</br>
-Is the study design appropriate to address the stated objectives?</br>
-Is the population clearly described and appropriate for the hypothesis being tested?</br>
-Is the sample size sufficient to ensure adequate power to address the hypothesis being tested?</br>
-Were correct statistical analysis used to support conclusions?</br>
-Are there concerns about ethical or regulatory requirements being met?</br></br>

Reviewer #1: Sound methodology.

The Abstract says new cases were analyzed, but this is not actually stated in the Methods section of the full paper. It would be interesting to know how many other cases were seen over this time period (previously treated, true relapses, people attending to continue treatment started elsewhere, etc.), although the subject of the paper is clearly limited to new cases.

**The study focusses on just new patients. This is stated on l 124 in the Methods section and in the objectives. We excluded patients who had been treated elsewhere l133. We do not have data on the other cases attending the clinic so this cannot be added.**

Reviewer #2: In this retrospective analysis, data was collected from 1995 to 2018 of patients diagnosed with leprosy at the HTD. Data on demographics, migration, travel, symptoms, diagnostics, treatment were collected and reported. The data is clearly presented in Tables (1: Sex and age at diagnosis, 2: Presumed country of acquisition, 3: WHO region of birth, 4: Clinic features of RJ Classification, 5: First referral specialty, 6: Time between arrival in UK and symptom onset). The methods are clearly defined and appropriate

**No response needed**
--------------------

<b>Results</b></br></br>
-Does the analysis presented match the analysis plan?</br>
-Are the results clearly and completely presented?</br>
-Are the figures (Tables, Images) of sufficient quality for clarity?</br></br>

Reviewer #1: The paper is about diagnosis, which is covered well.

One area I think could be expanded is the information about skin biopsies, which were done on 119 cases, but only 88 (74%) were diagnostic for leprosy. What were the results for the 31 cases that did not indicate leprosy? Were there other diagnoses, or were they mainly 'non-specific' changes? Another question of interest is whether the positivity rate changed over time - perhaps improving in later years, with better techniques, or perhaps declining due to loos of expertise? Were any biopsy results revised on further examination?

**The skin biopsies that did not contribute to the diagnosis could not show other diagnoses** because **this study had only confirmed new patients in it. The non-diagnostic biopsies showed non-specific changes of inflammation in the skin**. **Of these 31 patients with no histological features in their biopsies, 19 had neg slit skin smears, 7 had positive slit skin smears (range of BI 0.2-6)**

**The numbers too small in this study are to comment on trends about accuracy of diagnosis.**

**No biopsy results were revised.**

As the diagnosis in children is becoming a key indicator of transmission, I think it would be helpful to give more detail about the two child cases - where were they thought to contract leprosy? It would also be helpful to know the age of the persons diagnosed after living in Kosovo and Jamaica, which are probably now non-endemic - one would expect them to be quite elderly.

The child cases contracted leprosy in their home countries, Brazil 1, East Timor 1 and India (2) aged 13,14, and 18 (2). The **Patient from Kosovo was in his 20’s. l 200 states that his parents lived in from a former leprosy settlement, this is parents had leprosy.**

Reviewer #2: The results of the retrospective analysis are clearly presented in tables. The information is depicted in a clear and concise manner and each table is relevant to the study.

--------------------

<b>Conclusions</b></br></br>
-Are the conclusions supported by the data presented?</br>
-Are the limitations of analysis clearly described?</br>
-Do the authors discuss how these data can be helpful to advance our understanding of the topic under study?</br>
-Is public health relevance addressed?</br></br>

Reviewer #1: The Conclusions are sound.

Reviewer #2: This is an excellent and important retrospective analysis of patients with leprosy that presented and were treated at HTD from 1995- 2018. The authors discuss that the majority of the patients acquired leprosy in their country of birth "whether prior to arrival in the UK or on return visits". Given the timing of acquisition of leprosy, it was felt that leprosy is not a disease of travellers. Overall, the conclusions appear to be well-supported by the data. I appreciate the data on time between arrival in the UK and symptom onset. Interestingly, 26% had been present in the UK for greater than 10 years. I wonder if the patients with delayed symptoms visited endemic countries for prolonged periods of time allowing for acquisition. I appreciate the author's discussion regarding the potentially long incubation period, which may also explain this interval.

The data on nerve function and number of patient's in reaction at time of diagnosis is valuable as well when patients present with leprosy as a diagnosis.

Overall, this is an excellent and well-written manuscript that has organised and presented a large of amount data regarding patients with leprosy who presented to HTD. It is an important disease to recognize in patients from endemic regions.

--------------------

<b>Editorial and Data Presentation Modifications?</b></br><br/>
Use this section for editorial suggestions as well as relatively minor modifications of existing data that would enhance clarity. If the only modifications needed are minor and/or editorial, you may wish to recommend “Minor Revision” or “Accept”.

Reviewer #1: (No Response)

Reviewer #2: This is an excellent manuscript with a substantial amount of data that is very relevant to the field of infectious disease among other fields. I recommend minor revision. With these revisions, I recommend accepting this manuscript for publication.

--------------------

<b>Summary and General Comments</b></br></br>
Use this section to provide overall comments, discuss strengths/weaknesses of the study, novelty, significance, general execution and scholarship. You may also include additional comments for the author, including concerns about dual publication, research ethics, or publication ethics. If requesting major revision, please articulate the new experiments that are needed.

Reviewer #1: The paper is well presented.

Reviewer #2: This is an excellent, well-written and eloquent retrospective analysis on a large and import color of patients see at HTD from 1995-2018. I want to commend the authors for the amount of data obtained and analyzed. This study highlights several important factors including potentially long incubation time to develop leprosy, diagnostics, treatment with need for multidisciplinary care. The stigma of leprosy is also addressed, which is important. This is an important study and will be a valuable contribution to the literature. I have a few small recommendations.

line 42: please correct comma location: 53, 33.1%
line 272: MDT side effects mentioned. Are there any side effects of ROM that were experienced by patients?

No adverse effects of ROM recorded
Line 371: thalidomide is mentioned as a treatment. It would be interesting for the authors to list treatments that were used for type 1 reactions and ENL in addition to thalidomide. I do not think patient specific data is needed, just a general list of medications would be interesting.

Patients with Type 1 reactions were treated with a 32 week of steroids. Patients with ENL were treated with steroids and then Thalidomide.
